# From Darwin to the Census of Marine Life: Marine Biology as Big Science

**DOI:** 10.1371/journal.pone.0054284

**Published:** 2013-01-14

**Authors:** Niki Vermeulen

**Affiliations:** 1 Centre for the History of Science, Technology and Medicine, University of Manchester, Manchester, United Kingdom; The City University of New York-Graduate Center, United States of America

## Abstract

With the development of the Human Genome Project, a heated debate emerged on biology becoming ‘big science’. However, biology already has a long tradition of collaboration, as natural historians were part of the first collective scientific efforts: exploring the variety of life on earth. Such mappings of life still continue today, and if field biology is gradually becoming an important subject of studies into big science, research into life in the world's oceans is not taken into account yet. This paper therefore explores marine biology as big science, presenting the historical development of marine research towards the international ‘Census of Marine Life’ (CoML) making an inventory of life in the world's oceans. Discussing various aspects of collaboration – including size, internationalisation, research practice, technological developments, application, and public communication – I will ask if CoML still resembles traditional collaborations to collect life. While showing both continuity and change, I will argue that marine biology is a form of natural history: a specific way of working together in biology that has transformed substantially in interaction with recent developments in the life sciences and society. As a result, the paper does not only give an overview of transformations towards large scale research in marine biology, but also shines a new light on big biology, suggesting new ways to deepen the understanding of collaboration in the life sciences by distinguishing between different ‘collective ways of knowing’.

## Introduction

While the discovery of space is well under way and almost every piece of land in the world has been discovered and mapped, not much is known about the world's oceans that cover about 70% of the earth's surface. Especially life in the depth of the oceans and invisible life such as micro-organisms are still a big mystery. This inspired the ‘Census of Marine Life’ (CoML), a large-scale international research project that took place during the first decade of the new millennium. The collaboration did not only reveal micro-organisms, but also aimed to catalogue all the animals in the world's oceans, including life in the deep-sea “to assess and explain the diversity, distribution, and abundance of marine life in the oceans – past, present, and future” [1]. This means that the Census of Marine Life is part of a natural history tradition in which collaboration is necessary for the collection of research materials that are globally dispersed [2]–[3]. While the Human Genome Project (HGP) is often presented as the first large-scale research project in the life sciences, natural history shows that scientific collaboration is hardly new to biology. It is found already in the alliance between science and exploration that set out to map the world and collect and describe its diverse forms of life [4]. However, studies of scientific collaboration pay little attention to these collaborations that collect, identify and catalogue life. If field biology is gradually becoming an important subject of studies into big science [5]–[9] research into the world's oceans is not taken into account yet. This paper will therefore explore large-scale research efforts in marine biology further. Does CoML still resemble traditional collaborations to collect life, or have developments in biology research and recent changes in the relation between science and society transformed marine biology research?

Presenting marine biology as big science, the paper will start with an introduction into big science and the discussion on big biology. After an overview of the historical development of marine biology, it will present the Census of Marine Life as a contemporary example of such collaboration, showing transformations in marine biology. By discussing various aspects of collaboration, including size and diversity, internationalisation, research practice, technological developments, the application of research, and public communication, the paper shows how the exploration of life in the oceans started hundreds of years ago with relatively small forms of collaboration that developed over time, increasing in scale and scope while also transforming research practice. Contemporary developments in science and society have become integrated in the traditional natural history style of research, transforming the ways in which life is measured, mapped and modeled. By analyzing marine biology as an example of big science, this paper will not only give an overview of transformations in marine biology as a type of natural history research, but also shine a new light on ‘big biology’ and the ways in which large-scale collaboration in biology can be understood.

## Materials and Methods

The argument in this paper is based on an interdisciplinary study of scientific collaboration in biology, combining historical, philosophical and sociological perspectives [10] [11]. Next to an extensive analysis of existing theory on scientific collaboration, empirical research covered various contemporary large-scale collaborations in the life sciences, including the Census of Marine Life. This paper is therefore not a direct result of the History of Marine Animal Populations that is part of CoML and has as its main concern the reconstruction of human-nature relations over time and the exploration of historical exploitations patterns in marine ecosystems. In contrast, the paper shows the historical development of research into marine biology. Nevertheless, these two subjects are indirectly related, as research into marine life has been influenced by human-nature relations and has also played an important role in shaping those relations. In order to analyze the ways in which scientific collaboration in marine biology has transformed over time, the paper draws on conceptual analysis of the ‘big science’ concept and an analysis of literature on the development of ocean research and marine biology. To study CoML as a contemporary collaboration in marine biology, I used qualitative methods, including document analysis, interviews with key actors in the project, and attendance of CoML meetings in the period 2005–2010.

## Analysis and Results

### Big Science

The Census of Marine Life is part of a broader development towards large-scale projects in biology, also called ‘big biology [12]. The origin of the term ‘big science’ lies in the United States where physicist Alvin Weinberg coined the term in 1961 [13], while the concept was further developed by historian of science Derek de Solla Price in his book *Little Science, Big Science* [14]. Their work is part of a pile of books with the term ‘big’ in the title that all address growth as a distinctive phenomenon of modern society, covering big business, big government, big democracy, big school, big machine, big foundations and big cities [15]–[22]. Like all these ‘big books’, Weinberg and De Solla Price write about increasing dimensions full of wonder and admiration, but at the same time evaluate them critically. Growth is described as part of progress and an inevitable exponent of modern industrial society, while it is also seen as a source of problems. Thereby the books on bigness breath the ambivalence of the modern condition: “To be modern is to find ourselves in an environment that promises us adventure, power, joy, growth, transformation of big ourselves and the world – and, at the same time, that threatens to destroy everything we have, everything we know, everything we are” [23].

Accordingly, from its emergence the concept of big science has an ambivalent understanding of growth that is characteristic for the modern condition and which is still very much visible in the two opposing views on big science in the debate on big biology, that emerged together with the Human Genome Project and subsequent increases in the organization of biology. Proponents present large-scale science as the new and more effective way to perform research nowadays: “scientific leaders agree that collaborative projects can produce results that would be impossible for specialized individuals working alone to achieve” [24]. In contrast, according to opponents big biology industrializes, bureaucratizes and politicizes research and dilutes creativity. To illustrate, genome sequencing was portrayed as “massive, goal-driven and mind-numbingly dull” [25]. Molecular biologist Sydney Brenner even joked that sequencing is so boring it should be done by prisoners: “the more heinous the crime, the bigger the chromosome they would have to decipher” [26]. In these discussions, the term big science provided the discussants with a strong rhetorical sword, but they never explicitly reflected on the concept itself or the specific ways in which biology became big science.

Besides being normative, big science also developed empirical significance, starting with De Solla Price's book that studies transformation in science. Originally a physicist, Price became interested in the history of science, and the annual expansion of the Philosophical Transactions of the Royal Society triggered his fascination for what he later would call big science: “the piles made a fine exponential curve against the wall, I (…) discovered that exponential growth, at an amazingly fast rate, was apparently universal and remarkably longlived” [27]. This stimulated his work on the quantitative measurement of scientific development and scientometrics [28]. In addition to big science being a quantitative empirical phenomenon, the concept is connected to qualitative studies of scientific transformation. Against the background of the development of Science and Technology Studies, big science has been used to look into historic and contemporary practices of research collaboration. Detailed case studies of different forms of big science in fields as diverse as astronomy, ecology, physics and space research enriched the empirical understanding of big science [29]. The emergence of large-scale research complexes is perceived as a broader trend and common features are not only found in growing numbers but also in large, expensive instruments, industrialisation, centralisation, multi-disciplinary collaboration, institutionalisation, science-government relations, cooperation with industry, and internationalization. Themes that also feature prominently in more recent studies of scientific collaboration [12] [29] [30].

As a result, the big science concept should be seen as a historic concept that was formed in the 1960s to reflect on increasing dimensions in science, while acquiring different meanings over time: the big science concept has an empirical as well as an evaluative side. Moreover, when looking at big science empirically, a division can be made between a quantitative and a qualitative perspective and when using the concept to evaluate, positive as well as negative views on big science can be distinguished. Remarkably, discussions on big biology do not reflect on these different meanings, nor use the empirical side of the concept to investigate what kind of transformations actually take place in biology. Moreover, this disconnection results into an exclusive concern with the attributes of bigness, drawing attention away from “the more significant and interesting question of how science becomes larger” [31]. It is this process of making science big that I have called the ‘supersizing of science’ and the Census of Marine Life is an excellent example of the expansion of marine biology research.

### Marine biology as big science

Although particle physics and space research are identified as typical forms of big science with gravitating activity around large-scale technology, it is biology that has the longest tradition in scientific collaboration all be it on a smaller scale. Natural historians were part of the first forms of scientific collaboration, described as the ‘grand alliance’ between science and exploration in the 17th century. Traditionally, natural history research took place in the context of the Renaissance, figuring trading nations, empire building and the establishment of scientific societies and national museums and the most important reason for cooperation in biology was the dispersed character of biological material [2] [3]. Natural historians joined expeditions exploring the unknown world in order to describe, collect and catalogue new species, accumulating facts about plants and animals. In 1600 only around 6000 plant species of plants were known; by 1700 botanists had added discovered 12,000 new species, with similar accumulations in zoology. This advanced classificatory schemes– leading to Linnaeus's Systema Naturae [32] and the evolutionary theories of Lamarck [33] and Darwin [34] – but also changed in significant manners the ways in which biologists related and communicated with one another and acquired their research materials; infrastructural developments in transportation and communication technologies were crucial for these first forms of collaboration.

The great scientific voyages of the 18th and 19th centuries – including Charles Darwin's famous journey aboard the HMS Beagle – only explored species near the surface of the ocean, because they had neither access to nor knowledge of the deep oceans. For a long time it was thought that life could only be found there and at the ocean surface, as the absence of light, low temperatures and the density of water in the deep ocean was assumed to prevent life. However, these ideas slowly changed with the development of technologies that made the deep ocean visible [35]–[37]. At first, scientists began to investigate the depth of oceans. Sound to measure depth was first ventured by the Swiss mathematician Colladon in the Lake of Geneva, using a church bell and an ear trumpet. In 1838 this method was transferred to the ocean using explosions. In 1853 this developed into the so-called ‘soundingline of Brooke’ which was employed by the US Navy Depot of Charts and Instruments to map the North Atlantic Ocean. Thereby they discovered the ‘telegraphic plateau’ between Newfoundland and Ireland, which would be used by the Atlantic Telegraphy Company for the first trans-oceanic cable in 1858. In the 1870s the crew of the converted warship Challenger – known as the mothership of oceanography – discovered the Mid-Atlantic Ridge. As a result, scientists slowly began to realise that the oceanfloor had similar characteristics as the earth's surface, and in 1904 the newly established International Hydrographic Bureau published the first bathymetric standardised chart of the world ocean, based on 18400 soundings.

In the 20th century, ocean research gradually professionalized [38]–[40]. Next to telegraphy, shipping traffic, and the Titanic disaster, the World Wars and the following Cold War were important incentives to develop new technologies to survey the oceans and ocean research became institutionalised. To illustrate, the in 1930 founded American Woods Hole Oceanographic Institution (WHOI) played an important role in the development of oceanography. The history of the research vessels used by the institute gives a nice overview of the evolution of research vessels from traditional small sail and steamer ships to the modern big research vessels that are used today [41]. From the 1960s onwards, marine science increasingly became an academic endeavour and the 1970s were even pronounced to be the decade of ocean research. As a follow-up of the International Geophysical Year, the 1970s were arranged to be the International Decade of Ocean Exploration (1971–1980), aimed to scale-up ocean research to an international level [42].

As marine biology developed in close interaction with these more general explorations of the oceans, actual observations of life in the deep-sea are of fairly recent date [35] [36] [38]. In the 19th century the Irishman Forbes developed and professionalized the art of dredging in order to explore life in the deep. Later, scientists from the Woods Hole Institute combined a dredge and trawl into a so-called ‘epibenthic sled’, used for bringing the diversity of life in the deep oceans to the surface. In the course of the 20th century, scientists have increasingly gained access to the deep ocean, facilitating direct observation of life in the deep-sea. The development of the ‘bathysphere’ – a kind of underwater balloon – in the 1930s enabled the first observations of life. Later, in the 1960s, the ‘bathyscape’ – a kind of underwater zeppelin for two men – descended to the Challenger Deep of the Mariana Trench (at about 11 kilometres the deepest known spot in the oceans). They spend 20 minutes there and saw a fish, which indicated that even in the deepest ocean life is possible. The construction of the submersible with robot-arms ‘Alvin’, in 1964, gave researchers even better access to the depths of the ocean, as did the development of deep-sea cameras. In short, the investigation of oceanlife developed through interaction between scientific curiosity, societal exploitation of the sea and technological developments.

Investigations into the oceans and their living creatures is big science avant la lettre. “Marine studies in general have a very early history in collaboration: it is essentially big science” [43]. In marine biology, large-scale collaboration is not only stimulated through the globally dispersed nature of the research material but also through a multi-disciplinary approach: “The multidisciplinary character of problems asks for collaboration between for example biologists, physicists and chemists” [44]. Moreover, technology is a reason to collaborate as costs are high: “The instruments of marine science can be compared to the large and expensive instruments that are used in physics and astronomy, like huge telescopes and cyclotrons (…) for instance a research vessel costs about $60 million” [45]. However, attention and funds for this branch of science is still very small compared to, for instance, research into space [46] [45]. Nevertheless, today's debates on climate change and biodiversity have granted more prominence to ocean research and efforts such as the Census of Marine Life.

### The Census of Marine Life

Taking the collection work of Charles Darwin and his fellow natural historians some big steps further, the Census of Marine Life aimed to make an inventory of all animal life in the oceans –from the ocean shores to the deep-sea and from the poles to the Caribbean. CoML was put together at the end of the 20^th^ century, lasted 10 years (2000–2010), and involved 2,700 scientists, 80+ nations, 540 expeditions, US$ 650 million [47] [48]. It resulted in 2,600+ scientific publications, 6,000+ potential new species, 30 million species records and results are still being produced. The story goes that the project started during holidays at the seaside, with two men - Fred Grassle, a professor in benthic ecology at Rutgers University, and Jesse Ausubel, programme officer with the Alfred P. Sloan Foundation and professor in human ecology at the Rockefeller University in New York - meeting over a beer and discussing the possibilities to put more focus on biodiversity. They came up with the idea of counting the ocean's fishes and started to set-up the project that later became known as the Census, in interaction with the marine biology community and with the support of funding for coordination of research from the Sloan Foundation. CoML comprised seventeen global projects. First of all, fourteen field projects mapped current life in the oceans, varying from the deep-sea to the shores and from Antarctic life to coral reefs. The results were catalogued into a database by an overarching project. Finally, two projects studied respectively the past and present of life in the oceans: the History of Marine Animal Population and the Future of Marine Animal Populations. As a result, the Census existed of a patchwork of projects that was held together by a central governance structure: a Scientific Steering Committee with a secretariat, as well as regional nodes.

With its objective to catalogue life in the oceans, the Census of Marine Life could be defined as a form of contemporary natural history collaboration. The project especially enabled the making of connections, thereby transforming the life of the scientist involved: “The programme is about the connections (…) The Census is only possible if you are a community and you share the same language and the same world” [44]. Connections made within the Census were geographical – as it brought researchers from diverse countries together – as well as epistemological, as it brought disciplines together in a multi-disciplinary effort, and fostered diverse research questions and approaches. Connections were made on the governance level and in the various research parts of the Census. Although the scientists within a project often already knew each other, the collaboration developed the contacts:

You are able to work with the same samples, with the same goals. For example, we work together with a large group on zooplankton and we worked together on the cruise to gather the samples and now we are also going to work together in the lab to analyse the samples. In this way you can sort things out together and discuss strange things you encounter. (…) in this way the relationships become clearer, you have more insight in the connections. [49]

In other words, within the Census colleagues became collaborators, enlarging the knowledge of biodiversity within the oceans ecosystems. However, when comparing it with earlier forms of collaboration to collect life, it was also larger, profiting from scientific and technological advancement, transforming research practice and results (e.g. virtual database, modelling), while emphasizing application and public communication.

#### Expansion

Although natural history has been a collaborative effort from its start, the Census of Marine Life had unprecedented global ambitions, covering all the world's oceans as well as the diverse areas within these oceans, within the 14 field projects. While the Census started out as an American initiative, it became an international endeavor with over eighty countries participating. First, it stimulated cooperation between the United States and Europe: “This kind of collaboration has great additional value as people in Europe and the US have different specializations that we can now bring together which gives us new insights” [49]. And after covering the East and West Atlantic, the Census soon spread towards other regions as well: “Many countries, including India and China, have strong research programmes in marine biodiversity, which should enhance the longer term focus on Census related issues” [43]. Global expansion was supported by the creation of regional and national nodes in amongst others Australia, Canada, the Caribbean, China, Europe and the Indian Ocean. And next to space, time was an important dimension in the expansion of CoML. While the project itself took 10 years, its research intended to cover past, present and future, explicated in the three overarching research questions: what lived in the oceans, what lives in the oceans and what will live in the oceans? For answering such broad and complex questions a global collaborative effort was a requirement. And although the project's goal of counting and mapping all animal life in the oceans was clearly not reachable within one single decade, the final meeting of the Census in October 2010 presented many findings as well as some plans to extend the project into the next decade.

#### Technology development

Building on the history of ocean research, the Census made use of the most advanced technologies, and developed them within special technology working groups. Technologies were related to various research practices and stages. For transportation the research vessel was the most important technology, but also, helicopters and planes were used, for instance to access remote areas or to study whales. For underwater exploration manned submersibles, remotely operated vehicles (ROV's), autonomous underwater vehicles (AUV's) and Deep-Towed Vehicles (DTV's) were used. Next to technologies for transport, the Census employed technologies for observing, counting, collecting and studying movement: acoustic technologies (such as sonar and echo) and optical technologies (e.g. cameras, videos, lasers, satellites, microscope). The collection of samples took place with the help of (traditional) fishnets, trawlers, sledges, bottles, traps and by hand. Finally, the movements of fish was studied with the help of fishnets, satellites, sonar, echo and the tagging of fish. For example, the website of the TOPP project (Tagging of Pacific Predators) followed the movements of tagged predators such as sharks, turtles and elephant seals. Within the research projects scientists experimented with the use of these various technologies: “It is really good that attention is given to technology. On the one hand attention is given to technologies and expertise that is already available within the project, and on the other hand new opportunities are explored” [49]. Technologies enabled new visions of life and transformed research configurations, through the transformation of the spatiality of the research situation, the place of action and the area of attention.

#### Reinventing taxonomy

The transformation of research practices in interaction with developments in technology could also be seen in the case of taxonomy: the identification of species that is fundamental to natural history. Although it was a crucial practice within the Census and biology at large, it was and still is extremely difficult to find funding for taxonomic research. Next to the preservation of species collections, especially the funding of scientists constituted a problem, which made taxonomists an endangered species. As a result the Census focused on the development of technologies to determine species: “They explore if there are other possibilities than the traditional labour intensive determination using a microscope” [49]. Especially, the integration of genetic technologies within taxonomic practices was an important issue and the Census set up a DNA working group, which gave birth to the barcoding of life initiative. In analogy with using barcodes to identify manufactured goods, the DNA barcode initiative wanted to enable the identification of species by sequencing a uniform target gene, either in the laboratory or through a kit that could be used in the field [50] [51]. On the one hand the use of DNA to identify species enhanced taxonomic practice, and enabled the identification of species that could not be identified by traditional taxonomic methods, such as micro-organisms that account for more then 90 percent of oceanic biomass, or creatures from the deep sea which are often damaged as a result of changes in pressure. Moreover, genetic information played an important role in determining the relation between different species, and enabled the identification of new species and the relationship between species. On the other hand the use of genetic technologies did not really replace old-fashioned taxonomy, as the making of the barcode system required taxonomic expertise and the barcoding did not always work in practice: “For some fishes it works and for others it doesn't” [44]. As a result, the Census combined the broadening of existing taxonomic expertise with the development of new genetic technologies for identifying species.

#### Building a new information infrastructure

In natural history collaboration, data about species are always the main result of research. The way in which these data are assembled, standardised, integrated and stored is crucial, not only for the research practice, but also for the future outlook on life [52] [53]. Therefore, developments in information technologies transform the way in which data are stored, creating new memory practices. This also became apparent in the Census of Marine Life that has developed its own database called OBIS, which stands for Ocean Biogeographic Information System [54]. “OBIS lets you trawl 12 marine databases for collection records” [55] and has continuously expanded. OBIS performed an important role in the formation of the collaboration, and it collected the various research results, making them freely available on the internet. The socio-technical connections that made up OBIS integrated the diverse research projects and underpinned the collaboration that investigated life in the oceans. More specifically, the database combined two types of information: information on living organisms (taxonomic databases) and geographical information (GIS), displaying where species have been found. It is important to note that data sharing has been an essential part of OBIS from its start and the open-access database has become the lasting legacy of CoML provided that it will be continuously maintained and updated. In addition, CoML stimulated open-access publishing, encouraging researchers to publish in the journals PLoS Biology and PLoS One, creating CoML Collections related to results from the diverse projects (http://www.ploscollections.org) and collaborating towards an open-access Biodiversity Hub (http://hubs.plos.org/web/biodiversity). As such CoML was one of the first big science projects that emphasized the importance of global access to research data and results, setting an example to the wider scientific community to commit to open-access publishing.

#### Tracing the past and modelling the future

While natural history research has always served as a basis for learning and theorising about the development of life, this mainly concerned the evolution of life. In contrast, CoML aimed to use historic and contemporary data to explicitly learn about the future of ocean life. First, the History of Animal Populations project reconstructed direct human-nature relations over time, for instance through historic records of fish and the study of fish availability and prices on old restaurant menus. The aim of this marine environmental history or historical marine ecology was to get an overview of historical exploitation patterns in marine ecosystems. Through combining data on ocean life in the past with contemporary research data, CoML explicitly aimed to learn about the future. Therefore the Future of Marine Animals Project (FMAP) developed models to interpret historical data, designed field studies, synthesized data and made predictions about the oceans of the future. FMAP produced some interesting results, most notably a prominent publication in *Science* [56] on the downward trend in the diversity of fish in the open ocean due to fishery activities. By comparing information on the number of tuna and billfish caught on a standard longline with 1000 hooks from 1952 to 1999, the authors put together an overview of the decrease of fish in the open ocean, resulting in a striking visualization of a downward trend of 50%, coinciding with the emergence of large-scale commercial fishing. As this has serious consequences for marine biodiversity at large, the study resulted in global news coverage and gave rise to policy discussions. This is not to deny that Census scientists were struggling to fulfill their promise to predict the future of ocean life. The modelling of life in the oceans proved to be a real challenge, because the modeling efforts were relatively small and there was also not a proper picture of past and present ocean life in order to design a future model. Moreover, the Census scientists experienced that models cannot handle the complexity and unpredictability of ecosystems, as models can only contain a limited number of state variables, while ecosystems contain enormous amounts of species.

#### Application of research

While the application of research is not the primary goal of natural history research, the Census scientists experienced a clear shift in research policy from fundamental towards applied research. Although the Sloan Foundation recognized the value of fundamental research and supported it, other funding sources simply did not fund this kind of research and required applications. The relevancy of marine biology has from the 1970s onwards been found in environmental problems developing from pollution to climate change and biodiversity. A good example of an environmental application is the use of newly discovered marine microbes to solve ‘challenges’ concerning energy production, global climate change mitigation and environmental cleanup. In addition, research within marine life had some concrete (industrial) applications, such as technology development in the areas of information technology, the tracking of organisms, satellite connections, online observatories and genomics. In analogy with space research, marine science also helped to develop new materials, for instance isolation material, and underwater circumstances provided knowledge about what happens with life at low levels of oxygen. Finally, funding organisations often stimulated collaboration with industry in order to apply research. For marine science, this involved an array of companies and business activities, ranging from aquaculture or fisheries to instrument makers, and the pharmaceutical and energy industry. However, the most important application of CoML might well be found in its policy advice. Although not anticipated by the scientists from its start, the Census has contributed to the development of policies related to marine observation, planning and protection [48].

#### Showing the public

Since the emergence of (public) aquariums, Jules Verne's *Vingt mille lieues sous les mers* [57] and the movies of Jacques Cousteau, the underwater world has been a public attraction. In line with this tradition, the Census provided a new impetus to the public's awareness of life in the oceans, and thereby it also reflected the current trend towards the embedding of science in society. “The oceans, like the heavens, offer a preferred route to increasing public understanding of the world in which we live, and of science” [58]. According to the Sloan Foundation researchers should share what they do with the society: “Sloan does not think of ‘public relations’. Sloan seeks to advance both the scientists' understanding of the public and the public understanding of science” [59]. Consequently, the international secretariat of the Census developed a communication strategy – which resulted in frequent worldwide newspaper coverage – and all projects were required to pay attention to interaction with the public. Also the web was an important part of CoML, with a main portal giving general information on the Census and an introduction to its different components while each project had its own website with detailed information on research plans, activities and outputs. On top of making public communication daily business, various special initiatives were developed, including several books on life in the oceans, a travelling exhibition called ‘Deeper than Light’, and an Ocean movie directed by Jacques Perrin who made successful documentaries on monkeys, insects and birds before. Last but not least, the Census projects and scientists were involved in educational activities, making children aware of the importance of our living environment and stimulating them to choose a career in science. As a result, CoML has build on the public fascination for ocean life and expanded it further using both traditional and more modern forms of public communication.

## Conclusions

### New Natural History

According to sociologist of science Arie Rip [60] the ‘new natural sciences’ are still measuring, mapping and modelling the world, as the natural sciences always did, but now in a more sophisticated way, due to developments in information and communication technologies. In line with this argument, my analysis of transformations in marine biology collaborations – which can be seen as a form of natural history – has articulated issues of continuity and change. Continuity can be seen in measuring and mapping which was also the very design of the Census project. For one thing, the scientists named their project ‘Census’: it was about counting and mapping what populates the sea. And during one of the initial meetings of the Census, the project was presented as part of the exploration of the world: “The age of discovery is not over. Indeed, the voyages of discovery open to Charles Darwin, Captain Cook, and the explorers of Linnaeus' century are very much open to the voyagers of 2000 and beyond” [61]. However, the Census also showed how research has changed substantively, not only through ICT but in interaction with recent scientific, technological and societal developments. Together, these transformations reinvented marine biology as a form of natural history, making up what we may call new natural history.

To start, the scale and scope of marine biology is becoming ever larger. With the participation of more than 80 countries CoML aimed to cover all the worlds' oceans, broadening the scope of research geographically. As a result, marine biology has basically become a global effort. Next to this globalization, taxonomic research – a vital part of natural history – has transformed fundamentally. Where taxonomists traditionally used morphology to identify species, now a shift took place towards genetic identification, broadening the biological scope of the research, including the animals of the deep-sea and the world of micro-organisms. In addition, the integration and contextualisation of knowledge can be observed. Although identification and cataloguing of species was central, this was increasingly presented as a starting point for the creation of new knowledge through the integration of data. The inventory of ocean life was a tool that could be used in further research on the interaction between species and their environment: “We have to start with an inventory of good quality and you may then really focus on questions to explain relationships within biology” [44]. This increasing focus on ecosystems meant the integration of information about life and geography, which became visible in OBIS and modelling initiatives that contextualised knowledge about life and looked at its development over time. Finally, technological development and new relationships between science and society transformed research practices. The examination of the Census showed how the development of new technologies was part of changing research configurations that brought new visions of life. This could not only be seen in the transformation of taxonomic practices through genetic technologies, but also in the widening of observation through satellite technology and the building of the new information infrastructure OBIS, creating a new outlook on life in the oceans. Developments in the relationship between science and society were reflected in increasing attention to public communication and the application of marine research.

Moreover, the analysis of the Census showed how new natural history comes with its own particular problems. While the process that Rip [60] calls ‘sophistication’ implies that measuring, mapping and modelling practices are now more advanced and maybe even more effective, CoML put some major problems in today's marine biology forward. For instance, the use of genomics technologies for identifying species did not seem to solve the shortage of taxonomists and gave rise to controversies about ‘proper’ taxonomy. In addition, tensions between an international research scope and national funding structures were an important bottleneck for collaborative research, as was true of the lack of international governance structures geared to stimulating and regulating international ocean research. This caused that the limits of growth in marine biology collaborations became apparent: not all countries participated and not all species were catalogued. And finally, the Census of Marine Life struggled with the integration of all the available research material and the building of models. However, despite of relatively short-term funding cycles, the project also underscored the remarkable resilience of big science [62], as it seeks to extend itself into the future to eventually accomplish its goals.

### Reflections

In their characterisation of the big science concept, Capshew and Rader [31] present growth as the most important aspect of science: “the growth of science is perhaps its most notable historical characteristic, whether considered in terms of scope, scale, complexity, or impact”. While the discussions on big biology emphasized growth in the context of the Human Genome Project, this analysis of collaboration in marine biology as a form of natural history places these discussions in a broader context. When concentrating on growth in marine biology, it becomes clear that the exploration of life in the oceans started hundreds of years ago with relatively small forms of collaboration that developed over time, increasing in scale and scope while also transforming research practice. Contemporary developments in science and society have become integrated in the traditional natural history style of research, transforming the way in which life is measured, mapped and modelled. So although the Human Genome Project might be the first form of big biology in laboratory biology, it has been preceded and accompanied by increasing collaboration in field biology.

This analysis therefore suggests that when talking about big biology, and in order to come to a nuanced understanding of transformations in the organisation of life sciences research, different forms of collaboration in biology have to be taken into account. Such difference can be made through the contrasting of field and laboratory biology, or by looking into different sub-disciplines of biology, e.g. ecology, molecular biology, etc. However, through the notion of different ‘ways of knowing’ Pickstone [3] provides another way to make a distinction between different types of research in biology. While taking natural history as a starting point, he shows how an emphasis on the collection of species in cabinets and museums, gave way to times in which analysis and experimentation became central, together with the emergence of the laboratory as main research site. Distinguishing ways of knowing has the advantage that it connects epistemological and organisational perspectives on research, and can thereby also be used to explicate ‘collective ways of knowing’. These collective ways of knowing attend to various ways of collaborating with different timelines, as becomes visible when comparing for instance collaboration in natural history with more analytical oriented projects in laboratory biology (e.g. the Human Genome Project). Or one could compare different types of natural history collaborations, such as the Census of Marine Life with the Long Term Ecological Research network that monitors and compares life at various sites in the Unites States and Europe.

Next to identifying various forms of collaboration in biology, analysing collective ways of knowing allows for the description of scientific and organisational developments within specific ways of knowing, thereby also showing their interaction and entanglement. For instance, the Census of Marine Life does not only illustrate the development of collaboration in marine biology, but also shows its entanglement with analysis and experimentation in molecular biology through the ways in which more recent developments in laboratory biology transform the identification of species. Moreover, this perspective makes a comparison with collective ways of knowing outside of biology possible, as this typology of collaboration goes beyond the life sciences. For instance, when comparing contemporary large-scale projects in molecular biology and particle physics, a similar focus on the analysis of, and experimentation with, the essential building blocks of respectively life and matter becomes visible. While at the same time a difference in the size of technologies can be noticed, with implications for the organisation of research: while projects in physics centralise around large instruments, projects in molecular biology have a more decentralized character using ICTs to connect the different research sites.

In sum, the analysis of transformations in marine biology speaks to discussions on big biology. It is important to go beyond the polarisation of opponents and proponents of large-scale biology in order to understand the complexity of the transformations in the organisation of the life sciences. Not only the different meanings of big science, but also the different manifestations of collective ways of knowing in biology require attention. When analysing specific ways of knowing life, the complexity of scientific and organisational developments becomes clear, showing how the increase in scale comes in various forms and with different timescales. This more dynamic view on large-scale research, opens the way to a better understanding and a more nuanced outlook on big biology and its diverse manifestations.
